# Draft Genome Sequences of Two Novel Salmonella enterica subsp. enterica Strains Isolated from Low-Moisture Foods with Applications in Food Safety Research

**DOI:** 10.1128/genomeA.00183-18

**Published:** 2018-03-29

**Authors:** Devon R. Radford, Carlos G. Leon-Velarde, Shu Chen, Amir M. Hamidi Oskouei, Sampathkumar Balamurugan

**Affiliations:** a Guelph Research and Development Centre, Agriculture and Agri-Food Canada, Guelph, Ontario, Canada; b Agriculture and Food Laboratory, Laboratory Services Division, University of Guelph, Guelph, Ontario, Canada; c Agri-Neo Inc., Etobicoke, Ontario, Canada

## Abstract

The genomes of two strains of Salmonella enterica subsp. enterica serovar Cubana and serovar Muenchen, isolated from dry hazelnuts and chia seeds, respectively, were sequenced using the Illumina MiSeq platform, assembled *de novo* using the overlap-layout-consensus method, and aligned to their respective most identical sequence genome scaffolds using MUMMER and BLAST searches.

## GENOME ANNOUNCEMENT

Salmonella enterica is a leading cause of bacterial foodborne illnesses worldwide (1). Multiple outbreaks of Salmonella infection have also been linked to low-water-activity (a_w_) foods, such as nuts and seeds (2). However, the mechanisms utilized by this bacterium to ensure their survival in dry conditions have not been fully elucidated (3). We present here the draft genome sequences of two novel strains of S. enterica subsp. enterica: strain 17-030379-0001, isolated from dry hazelnuts (a_w_ = 0.483 ± 0.003) and serotyped as serovar Muenchen with antigenic properties 23:z29, and strain 17-030379-0002, isolated from chia seeds (a_w_ = 0.601 ± 0.003) and serotyped as serovar Cubana with antigenic properties 6,8:d:1,2.

Genomic DNA was extracted from an overnight culture using the DNeasy blood and tissue kit (Qiagen, Mississauga, ON, Canada), following the manufacturer’s protocol. Sequencing libraries were prepared from the DNA using the Nextera XT DNA library preparation kit (Illumina, San Diego, CA, USA), following the procedures described in the kit’s reference guide. Sequencing was conducted using a MiSeq sequencer with a MiSeq V2 reagent kit (Illumina) and 2 × 250 paired-end cycles, according to the manufacturer’s protocol. Raw sequence reads were filtered using the MiSeq sequencer system software (Illumina) to remove low-quality sequences and trimmed to remove adaptor sequences. Sequences passing a quality score of 30 were assembled *de novo* following an overlap-layout-consensus method using GeneStudio. In the case of the serovar Muenchen genome, this yielded 33 contigs for a total of 4,795,679 bp with a GC content of 51.29% at 1× coverage. The assembled serovar Cubana genome yielded 71 contigs for a total of 5,041,200 bp with a GC content of 51.3% at 1× coverage. The scaffolds of these genomes were mapped by alignment to S. enterica subsp. enterica serovars Yovokome (GenBank accession number CP019418) and Cubana (GenBank accession number CP006055) with 99.4 and 100% scaffold coverages, respectively. Assembly quality was evaluated using QUAST (4), which identified 3.37 mismatches/100 kb for the serovar Muenchen genome and 6.70 mismatches/100 kb for the serovar Cubana genome. MUMMER plots (5) were examined for contig internal inversions as markers of misassembly, and gaps were used to measure the extent of the genome coverage. Moderate gaps relative to the reference genomes were observed, but no evidence of misassembly was observed. Gene calling and annotation were carried out using the Rapid Annotations using Subsystems Technology (RAST) server (6). A total of 5,004 features were predicted in the serovar Cubana genome, falling into 578 subsystem categories, with 4,904 predicted coding sequences and 100 RNAs. RAST predicted 4,737 features in the serovar Muenchen genome, spanning 575 subsystem categories, with 4,649 predicted coding sequences and 88 RNAs.

These assemblies will allow researchers to further explore the genomic diversity of Salmonella serovars adapted to low-moisture environments, impacting basic and applied food safety research.

### Accession number(s).

The genome sequences reported here have been deposited at DDBJ/EMBL/GenBank under the accession numbers PJAL00000000, for Salmonella serovar Muenchen strain 17-030379-0001, and PJAK00000000, for Salmonella serovar Cubana strain 17-030379-0002.
